# Working Memory in Deaf Children Is Explained by the Developmental Ease of Language Understanding (D-ELU) Model

**DOI:** 10.3389/fpsyg.2016.01047

**Published:** 2016-07-07

**Authors:** Mary Rudner, Emil Holmer

**Affiliations:** Linnaeus Centre HEAD, Swedish Institute for Disability Research, Department of Behavioural Sciences and Learning, Linköping UniversityLinköping, Sweden

**Keywords:** deaf, sign language, working memory, language experience, vocabulary, cognition, imitation, representation

The ability to keep information in mind for processing is known as working memory and is vital for learning. Children who have difficulty keeping up in school may have working memory limitations rather than limitations in the specific tasks they are assigned. In particular, children with functional impairments at the sensory or cognitive level may have difficulty performing tasks, either because the impairment hinders the development of the working memory system as such or because it hinders the development of linguistic and cognitive skills underpinning working memory development.

Marshall et al. (2015) studied this issue by investigating working memory and its relation to language processing in two different groups of deaf children: native users of British Sign Language (BSL) and non-native BSL users, as well as in a control group of typically developing children with no hearing difficulties and no knowledge of sign language. The native signers had at least one deaf parent who had communicated in sign language with their child since birth. The non-native signers had acquired sign language later. All three groups performed two executively demanding non-verbal working memory tasks as well as an expressive vocabulary test and a narration task based on a filmed scenario enacted in BSL. Results showed that the non-native signers performed more poorly than the hearing participants on both working memory tasks while there was no difference in performance between the native signers and the hearing participants. The non-native signers had poorer vocabulary scores than the native signers who in turn had poorer vocabulary scores than the hearing children. However, there were no group differences on the narration task. Regression analysis showed that vocabulary was a significant unique predictor of performance on both of the working memory tasks. This association was all the more striking considering that there were no explicit demands on verbal skills in the working memory tasks. Marshall et al. (2015) argue that this pattern of results allows them to tease apart effects of auditory and language experience: while both of the deaf groups have experienced auditory deprivation, only one of them (non-native signers) has reduced language experience. In particular, the authors' interpretation is that while both auditory deprivation and reduced language experience have an impact on vocabulary development, reduced language experience, but not auditory deprivation as such, has an effect on the development of non-verbal working memory.

The connection between language experience and development of working memory is of both practical and theoretical importance. The interplay of linguistic and cognitive skills during language understanding under adverse conditions is described by the Ease of Language Understanding model (ELU, Rönnberg et al., 2013). This model proposes that when adverse listening conditions such as hearing impairment or background noise give rise to mismatch between the incoming language signal and cognitive representations, engagement of explicit working memory mechanisms is triggered. Such explicit processing is associated with a cost in terms of the cognitive resources that have to be diverted from other activities, such as learning. The ELU model has proved to have explanatory power for adults, but until recently it has not been applied to development.

In an experimental study conducted in Sweden (Holmer et al., 2016), imitation of manual gestures was elicited from deaf and hard-of-hearing (DHH) signing children and hearing non-signing children. The manual gestures used as stimuli belonged to three categories: lexical items in Swedish Sign Language (SSL, familiar to the DHH signing children but not the hearing children); lexical items in BSL (unfamiliar to both groups), and non-signs (phonotactically illegal and unfamiliar to both groups). Imitation of unfamiliar manual gestures can be compared to non-word repetition (Marshall, 2014), an established measure of working memory in the developmental literature (Gathercole, 2006). We hypothesized on the basis of the ELU model that pre-existing cognitive representations would allow the DHH signing children to imitate familiar signs more accurately than unfamiliar signs and that the performance of this group would be better than that of sign-naïve hearing children. We found no difference between groups on initial testing, although both groups imitated lexical signs more accurately than non-signs. However, when imitation was elicited a second time, we found that the performance of DHH signing children improved more than that of hearing children across gesture categories. Further, the second set of imitation scores was predicted not only by domain general language skills for both groups but also by modality specific language skills. On the basis of these findings, we proposed that both domain general and language modality specific skills are brought into play during working memory processing and that this leads to the establishment of new cognitive representations as well as to redefinition of existing representations (Holmer et al., 2016). This proposed mechanism represents a developmental extension of the ELU model, D-ELU (Holmer et al., 2016), see Figure 1.

**Figure 1:**
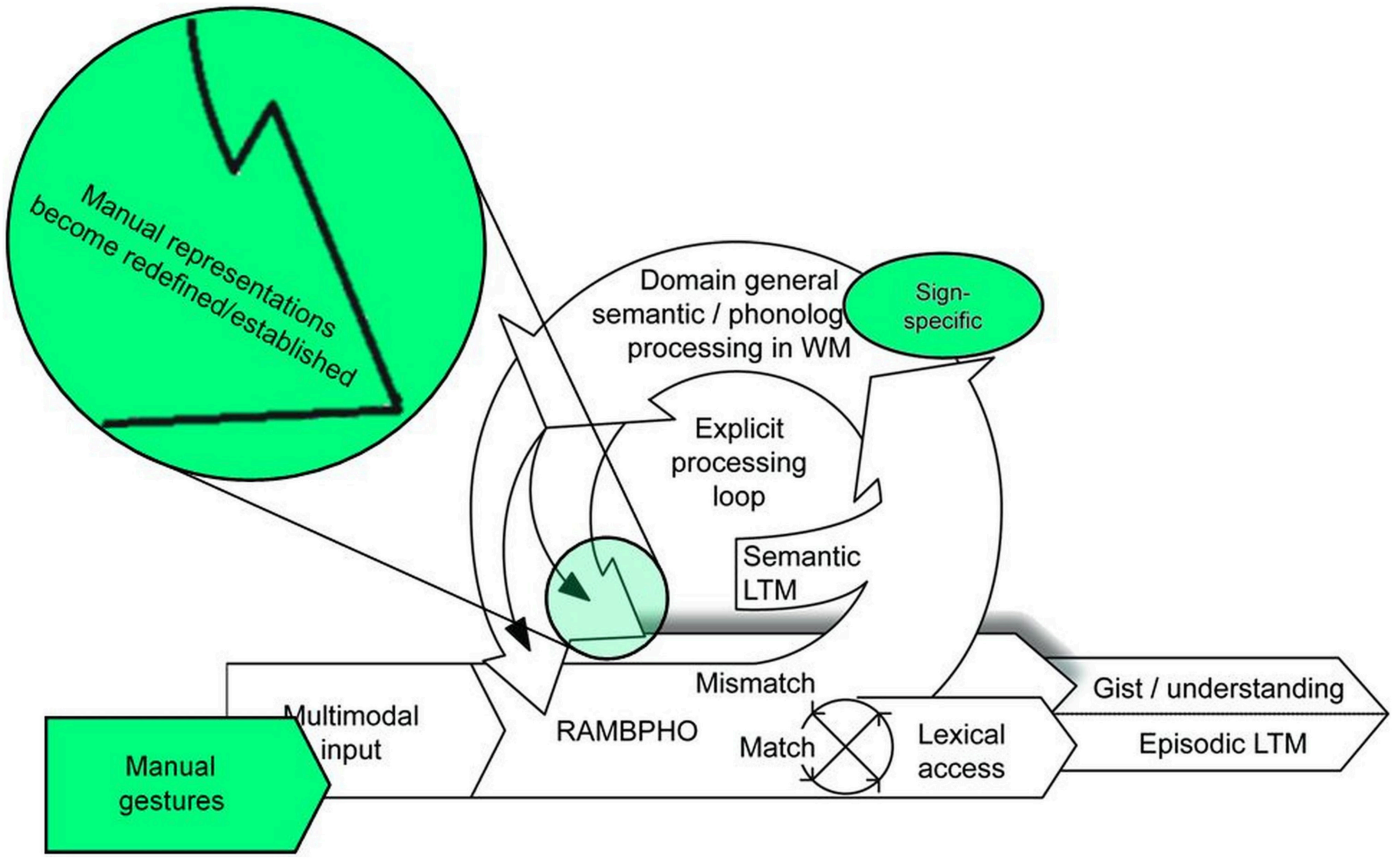
**The Developmental Ease of Language Understanding (D-ELU) model**. RAMBPHO stands for Rapid, Automatic, Multimodal Binding of PHOnology and LTM stands for Long-Term Memory. When input cannot be matched to long-term representations in the RAMBPHO buffer, an explicit processing loop is engaged that uses domain general and language-modality specific knowledge to redefine and/or establish appropriate representations in LTM. Adapted from “Imitation, sign language skill, and the Developmental Ease of Language Understanding (D-ELU) model” by Holmer et al. (2016). Copyright 2016 by Holmer, Heimann and Rudner under the CCBY3.0 license (http://creativecommons.org/licenses/by/3.0/).

The results reported by Marshall et al. (2015) can be understood in terms of the D-ELU mechanism. Although congenital or early deafness may lead to impoverished speech input, development of domain general working memory in DHH children can be supported by ensuring that they are engaged from an early age in executively demanding linguistic activity in an appropriate language modality.

## Author contributions

MR prepared the first draft of the manuscript. EH provided critical comments on the draft manuscript and MR and EH prepared the final version of the manuscript together.

## Funding

This work was supported by grant number 2008-0846 to MR from the Swedish Research Council for Health, Working Life and Welfare.

### Conflict of interest statement

The authors declare that the research was conducted in the absence of any commercial or financial relationships that could be construed as a potential conflict of interest.
